# Vasculitis in Africa

**DOI:** 10.1007/s11926-018-0711-y

**Published:** 2018-02-21

**Authors:** Eugene Genga, Omondi Oyoo, Adewale Adebajo

**Affiliations:** 1 Department of Clinical Medicine and Therapeutics, School of Medicine, College of Health sciences, University of Nairobi, P O Box 30197-0100, Nairobi, Kenya; 2 Department of Clinical Medicine and Therapeutics, School of Medicine, College of Health Sciences, University of Nairobi, P O Box 19676-00202, Nairobi, Kenya; 3 Faculty of Medicine, Dentistry and Health, University of Sheffield, Beech Hill Road, Sheffield, UK

**Keywords:** Vasculitis, Vasculitides, Africa

## Abstract

**Purpose of Review:**

Systemic vasculitides are characterized by inflammation of blood vessel walls leading to a myriad of organ disorders depending on the size, site, and location of the affected blood vessel. The epidemiology of vasculitis in the developing world has been inadequately documented. The description of the vasculitides in Africa, both from hospital series as well as taking into consideration, previous epidemiological studies in the community, indicates that these conditions have been rare until relatively recently. In view of these past observations, this review of publications on the topic looks to shed light on the current state of vasculitis in Africa.

**Recent Findings:**

Takayasu and Kawasaki appear to be the most commonly reported vasculitides in Africa. Most of the published reports are from North and South Africa. Furthermore, the contribution of vasculitis associated with infections, and in particular HIV, is significant.

**Summary:**

There are increasing numbers of publications reflecting a growing recognition of the vasculitides in Africa.

## Introduction

The vasculitides are a group of diseases characterized by inflammation in and around blood vessels and affecting various organs such as the kidneys, skin, joints, and lungs, among others. Vasculitis can affect both children and adults presenting in a variety of ways, from mild and transient symptoms to life-threatening ones. The vasculitides can be classified, according to the predominantly affected vessel, into small-, medium-, and large-sized vessel vasculitis according to the Chapel Hill Consensus Conference on the Nomenclature of Vasculitidies [1].

Until recently, the epidemiology of the systemic vasculitides has been poorly documented. It is now known that there are regional and ethnic differences in the clinical features of patients with vasculitis. Unfortunately, there is a paucity of data regarding these conditions in the developing world, including Africa, with most information arising from Western Caucasian populations. However, in recent years, there have been increasing reports which indicate that these conditions do occur in Africa, although hitherto infrequently reported. This review is intended to highlight recent reports of the systemic vasculitides from publications in Africa. Source data was obtained from searching relevant databases including PubMed, Medline, African Journals Online, and abstracts from the proceedings of conferences of the African League of Associations of Rheumatology (AFLAR).

## Large-Vessel Vasculitis

### Takayasu Arteritis

Takayasu arteritis is a rare, large-vessel vasculitis of unknown etiology that most commonly affects women predominantly below 40 years of age. Worldwide estimates are at 0.5–3 cases per million per year. The disease has a worldwide distribution although it occurs more commonly in Asia [2]. The vessels affected vary with geography, for example in Japan and Korea, the aortic arch is mainly affected while in India, Thailand, and Europe, the abdominal aorta appears to be more frequently involved. Most of the data from Africa has been published from Tunisia. Ghannouchi et al. in a review of 37 Takayasu patients from 1985 to 2005 noted a mean age at presentation of 33.2 years (range 16–68 years) and found that 88.9% were female. Intermittent claudication was the most common presentation (81.5%) and hypertension was noted in 40.7% of cases. The subclavian artery was the most frequently affected site in this cohort [3]. Another retrospective study done in southern Tunisia by Kechaou et al. between 1996 and 2006 collected and analyzed 29 patients (25 females and 4 males). The mean age at diagnosis was 35.4 years (range 18–65 years). Ninety-three percent of vessels affected included the aortic arch and its branches, while only 24% involved the renal arteries and 21% the abdominal aorta [4]. A retrospective study by Elasri et al. among 47 Moroccan patients reviewed between the years 1988 and 1999 reported that involvement of the aortic arch and its branches was more frequent than the involvement of the abdominal aorta and its branches [5]. Mwipatayi et al. also reviewed 272 cases from South Africa seen between the years 1952 and 2002. The mean age at presentation was 25 years (range 14–66 years) and 75% of patients were female. Interestingly, only 8% of the patients studied were Caucasian. Hypertension was the most common presentation (77%) and was usually a consequence of renal artery stenosis or aortic coarctation. The entire aorta was involved in 70% of cases. Thirty percent had aortic bifurcation involvement. The most common cause of death was cardiac failure [6•]. In addition, there have been occasional case reports of this condition, published from South Africa, Senegal, and Kenya [7–10].

### Giant Cell Arteritis

Giant cell arteritis (GCA) is a large-vessel vasculitis predominantly seen in those aged over 65 years of age. The distribution of GCA worldwide is not uniform with the highest incidence and prevalence rates occurring in Scandinavia, the UK, and Northern Europe. Incidence rates have been reported of 15–35 per 100,000 individuals aged over 50 years [2]. There are very few studies on giant cell arteritis which compare the condition in Caucasian and non-Caucasian populations. The conclusion of these studies has been that the incidence of this condition is much lower in the non-Caucasian populations [11, 12]. This may explain the low numbers of patients with GCA reported in Africa. Mabrouk Khalifa et al. [13•] reviewed 96 patients in whom GCA was diagnosed between 1986 and 2003 using the ACR criteria and reported a male to female ratio of 0.88 and a mean age at the time of diagnosis of 70.8 ± 7.7 years. The most frequent clinical manifestations were headache (91.7%), abnormalities in temporal arteries (85.4%), severe ischemic manifestations (80.2%), constitutional symptoms (75%), and polymyalgia rheumatica (56.3%). In another study from Tunisia, Lamloum et al. [14••] retrospectively reviewed patient hospital records over 30 years between 1986 and 2015 and found 90 patients with GCA using the ACR criteria. Of these 90 patients, 60 were men and 30 women with an average age at the onset disease of 76 years. There have also been occasional case reports of GCA from Egypt and Ethiopia [15, 16].

## Medium-Vessel Vasculitis

### Kawasaki Disease

Kawasaki disease (KD) is a medium-vessel vasculitis that affects mainly children under the age of 5 years, with an inclination for the coronary arteries. Kawasaki disease is most common in the Far East especially Japan, Korea, and China but has been found to be relatively uncommon in Africa, Europe, and the USA. The incidence of Kawasaki in Tunisia, Morocco, and Algeria has been documented as 0.95, 4.52, and 3.15, respectively [17]. Boudiaf et al. [18••] reviewed 133 children (82 boys and 51 girls) with KD that were seen at a public hospital in Algeria between 2005 and 2014. Of the 133 patients seen, 131 met the complete criteria for Kawasaki while two demonstrated incomplete criteria. The mean age at diagnosis was 31 months. The commonest signs were fever, rash, oral changes, and conjunctivitis [18••]. A study from Egypt reported that 6.7% of adults aged 40 years or younger and who underwent angiography for evaluation of possible myocardial ischemia had lesions consistent with antecedent KD [19]. A Congolese study reported on a case series of 11 patients with KD seen between 2003 and 2014, of which nine of these cases were classified as complete Kawasaki disease. The male to female sex ratio was 2.7 with a mean age of 16.5 ± 5.9 months with a range of between 9 and 43 months [20]. A review of 26 in-patients with a rheumatological diagnosis in a Kenyan pediatric hospital found that 4 patients (15.4%) had Kawasaki disease [21]. A Nigerian audit carried out on hospital patients seen between 2011 and 2016 identified eight cases of Kawasaki disease (four complete and four incomplete) with a male to female ratio of 3:1. The median age at diagnosis was 23.5 months. Coronary artery abnormality (CAA) was observed in three out of their eight patients or 37.5% of cases [22]. There have also been a few other patients with KD in Sub-Saharan Africa reported, notably from Ghana [23], Nigeria [24, 25], and Madagascar [26].

### Polyarteritis Nodosa

Kussmaul and Maier first described polyarteritis nodosa (PAN) in 1866. Following increased understanding of the vasculitides and the discovery of antineutrophil cytoplasmic antibodies (ANCAs) in the 1980s, the definition of polyarteritis nodosa has evolved over time. A negative ANCA differentiates PAN from ANCA-associated vasculitides as they have similar presentations pathologically and clinically. Depending on the definition used, PAN has an incidence of about 1.6 cases/million with a prevalence of about 31 cases/million in European countries [27]. The prevalence of PAN in the USA is between 3 and 4.5 cases per 100,000 population annually [28]. Data on the condition from Africa is surprisingly scarce, even though PAN has been associated with various infectious viruses including hepatitis B virus (HBV) and human immunodeficiency virus both of which have high prevalence rates in Africa. A study from Paris, for example, reported a prevalence of classical PAN of 30.7 per million people of which 30% were HBV positive [29]. In the same French cohort, vaccination with HBV vaccine led to the percentage of PAN patients with HBV decreasing to less than 5% [29]. Africa’s HBV and HIV endemicity makes it difficult to understand why there is an apparent low prevalence of PAN on the continent. Possible explanations include the fact that most African countries have overburdened health care systems as a result of numerous infectious diseases, and consequently are not geared towards identifying and managing the vasculitides. Associated with this problem is a lack of adequate laboratory infrastructure (including immunology facilities) and a shortage of appropriate medical personnel, among the various other challenges currently facing the African continent. There has been only one case of PAN documented from Africa. This was a 27-year-old South African male who was known to have diastolic hypertension and who presented with chronic urinary obstruction. A bladder neck mass was visualized on cystoscopy, and a biopsy confirmed a diagnosis PAN [30••].

## Small-Vessel Vasculitis

### ANCA Vasculitis

Worldwide, the incidence of ANCA-associated vasculitis (AAV) has been on the increase. This can be attributed to advances in diagnosis as well as to tools enabling better case definition, being available. AAV includes granulomatosis with polyangiitis (GPA), microscopic polyangiitis (MPA), and eosinophilic granulomatosis with polyangiitis (EGPA), all of which are readily recognizable. The prevalence rate in Europe has been reported as 20/million, with MPA being more common in Southern Europe, while GPA is more common in Northern Europe [2]. The incidence rates of AAV across various white Caucasian and non-Caucasian populations are different. Some studies have reported lower rates of AAV among non-Caucasians as compared to Caucasians [29]. Although this observation could explain the very low reported rates for these conditions in Africa, we believe that a combination of several factors is likely to be responsible. Such possible factors include a truly low incidence and prevalence of these conditions in Africa, a low index of suspicion for the conditions, and a lack of the appropriate laboratory facilities and relevant medical personnel. There are only very few studies on AAV from Africa. Between 2000 and 2012, a Tunisian hospital study [31•] found 21 patients with GPA. There were 14 men and 7 women with a mean age at diagnosis of 45.5 years (range 16–70 years). The major clinical presentations were 18 patients with ear, nose, and throat symptoms (85.7%), 16 patients with pulmonary involvement (76%), 12 patients with renal involvement (57%), 12 patients with mucocutaneous manifestations (57%), and 11 patients (52.4%) with neurological involvement. Between 2000 and 2014, another Tunisian study [32••] reviewed 30 patients with AAV. In their case series, there were 17 (56%) males and 13 (44%) females with a mean age at diagnosis of GPA of 46 ± 12 years. The major affected organs were ear, nose, and throat involvement (83%), lung involvement (70%), and renal (56%) involvement. Cytoplasmic pattern-antineutrophil cytoplasmic antibodies (ANCAs) were present in 27 (90%) patients. Eighteen patients had a favorable outcome while 12 patients died.

### IgA Vasculitis

IgA vasculitis which was formerly known as Henoch–Schönlein purpura occurs predominately in children. Its annual incidence in children in most studies range from 3 to 26.7/100,000 while in adults, the incidence ranges from 0.8 to 1.8/100,000 [33, 34]. This makes IgA vasculitis two to 33 times more common in infants and children than in adults. Data from Africa on this condition is also scarce, possibly due to under-reporting. However, the interpretation of the reported prevalence data requires consideration of the heterogeneous criteria used for the classification of this disease. This was evident with data from a Taiwanese study which indicated that almost 60% of children with IgA vasculitis were not admitted to hospital [34]. The majority of the surveys performed have been conducted by pediatricians, raising the possibility that cases managed by general practitioners or other community-based specialists were overlooked [35, 36]. This is likely to result in difficulties in identifying IgA vasculitis cases in the general population. In turn, this might impact on the number of cases of this condition, reported from Africa. A study from Tunisia [37] retrospectively reviewed 34 patients with IgA vasculitis who were seen between 1996 and 2010. The mean age at diagnosis of these patients was 7.23 years with an age range of between 3 and 14 years. Renal involvement was noted in 68.7% of cases. Another retrospective Tunisian study [38] reviewed patients seen between January 2005 and January 2014. The authors identified 24 children with Henoch–Schönlein vasculitis (HSP). There was a male predominance (sex ratio 1.8), with a mean age at diagnosis of 5.2 years. The main clinical manifestations were purpura (all cases), articular involvement (16 cases), and gastrointestinal involvement (11 cases). An Egyptian study [39] found that their patients with HSP had a significantly higher frequency of MEFV mutations (61.7%), when compared to the apparently healthy control population (36.7%). Two reviews of renal biopsies in Morocco identified 12 and 6.2% of their case series respectively, as having IgA nephropathy [40, 41]. There is notably a paucity of data on this condition among adult populations in Africa. A retrospective study undertaken in Morocco [42] looked at HSP in adults and identified 30 adult patients with the condition (18 women and 12 men), aged between 16 and 82 years. Vascular purpura was the most frequent initial clinical manifestation (90%). The other principal clinical presentations were inflammatory arthralgia in 56.6% of their patients, 23.3% with arthritis, while further 16 patients (53.3%) had gastrointestinal involvement.

## Other Vasculitis

### Behçet Disease

Behçet disease (BD) has been noted to have the highest occurrence worldwide along the old Silk Road, extending from the Middle East to China. The country with the highest prevalence of Behçet disease is Turkey at 420 cases per 100,000 population. The prevalence in Japan, Korea, China, Iran, and Saudi Arabia ranges from 13.5 to 22 cases per 100,000 population. The prevalence in North America and the other European countries is much less, with 1 case per 15,000–500,000 population [43]. Epidemiological data on Behçet’s disease in Sub-African population is scarce. Maodo Ndiaye et al. [44] documented 50 cases of the condition in an African black population from Senegal. The population comprised of 31 male and 19 female patients with an average age at diagnosis of 32 (18–67) years. Two patients had a positive family history of Behçet’s disease. The oral and genital aphthous lesions were present in all patients. The pathergy test was positive in 16 patients (32%). The authors noted, however, that the majority of the patients in their series initially received inappropriate treatment due to misdiagnosis, a consequence of the lack of knowledge of Behçet’s disease among the African general practitioners who first saw these patients. In East Africa, Éric Liozon et al. [45] documented a case series of 14 patients from the Comoros islands. The gender was predominantly male (ten patients). The authors also noted a delay in diagnosis of 5.5 (± 5.1) years. Five of their patients presented as neuro-Behçets, including three with progressive brainstem syndrome. Vascular disease was found in three patients, relapsing pan uveitis in two patients, and rheumatic symptoms with mucocutaneous disease in four patients. Thirteen patients were found to be HLA-B51 negative. The authors concluded that Behçet’s disease might be under-reported in Africa and that increasing awareness by physicians is needed so as to shorten time to diagnosis, as well as to improve the management of these patients. A study from Tunisia [46••] analyzed the demographic, clinical, and genetic features of 260 patients with Behçet’s disease. The cohort was predominantly male (male to female ratio of 2.61) with a mean age of onset of the disease of 29 years. Oral and genital ulcers were seen in 100 and 83% of their patients respectively. The other common clinical features of BD that they found were ocular involvement (51%), arthritis (38.8%), venous thrombosis (33%), and neuropsychiatric symptoms (24.2%). Only 1.5% had gastrointestinal lesions. HLA-B51 frequency was significantly higher in patients with BD than those in the general population and was associated with neurological symptoms. The authors concluded that there is a high frequency of deep vein thrombosis and neuropsychiatric involvement, but gastrointestinal lesions were rare in this population.

### HIV Vasculitis

Since the beginning of the human immunodeficiency virus (HIV) epidemic over three decades ago, more than 70 million people have been infected with the HIV virus and about 35 million people have died of HIV-associated disorders [47]. Sub-Saharan Africa remains the hardest hit region of the world, as over 70% of infected individuals reside in this region. The epidemiology of HIV-associated vasculopathy is yet to be fully established as most studies conducted have been hospital based and are consequently not a true representation of its prevalence in the general population. In addition, the exact pathogenetic mechanisms as well as the reasons for the varied manifestations of the HIV-associated vascular pathology are yet to be fully elucidated but would appear to be multi-factorial in etiology. Brand et al. [48] compared the histopathological features that characterize large vessel changes in HIV-seropositive patients as compared to HIV-seronegative patients with critical lower limb ischemia. They noted that HIV-positive patients were younger and had a lower prevalence of hypertension and diabetes mellitus than HIV-negative patients. Vasculitis in association with HIV can manifest in almost every pattern and type of blood vessel involvement. Robbs et al. [49••] described the presentation, management, and short-term results of therapy (less than 1 month) among patients admitted with HIV vasculopathy to a South African Hospital. There were 226 patients studied; 111 had aneurysms and 115 had occlusive disease. The study population was predominantly male (90%) and black African (98%) with an age at diagnosis ranging from 4 to 53 years (average age 36 years). The CD4 count ranged from 1 to 930 cells/mm^3^. A total of 202 aneurysms presented in 111 participants, of which the commonest sites were the superficial femoral artery (40%) and the carotid artery (25%). The occlusive arm of the study was divided into acute thrombosis with no previous claudication (51 patients) and atherosclerotic disease (64 patients). The majority of the patients presented with critical ischemia. Standard therapy was provided to all of the patients. The authors concluded that surgical therapy was beneficial in the short term for managing aneurysms and noted that for the surgical management of occlusive disease, there is a 25% overall salvage rate in the short term (less than 1 month). The longer term outlook for surgery however is uncertain. Otedo A.E.O et al. [50] also described vasculitis in African HIV patients and characterized their CD4 count levels, anatomical sites affected, and clinical patterns observed in two centers in Kenya. They studied eight patients (four males and four females) with an age range of 24 to 61 years (mean 33.13 years). The CD4 levels ranged from 2 to 200 cells/mm^3^. Two patients had coinfection with hepatitis B. Five patients had central nervous system vasculitis and three had peripheral vasculitis.

## Conclusion

Although Africa has one of the most genetically diverse populations worldwide, there remains a paucity of high-quality epidemiological data relating to the occurrence of the vasculitides on the continent. This is likely to relate to the heavy burden of infectious diseases and requiring attention, compounded by a host of socio-economic problems that are found on the continent. Despite this, it is encouraging to note that of recent, there have been an increasing number of publications from the African continent about the vasculitides. We believe that this reflects a growing recognition of the vasculitides by African physicians and improvement in the diagnosis and management of these conditions.

It is our view that these conditions are not rare in Africa but have hitherto been under-diagnosed. We believe that there will be increasing reports of these conditions in future, from the African continent.
